# Warning signs for identifying neurodevelopmental disorders: a systematic literature review

**DOI:** 10.1016/j.jped.2025.101478

**Published:** 2026-01-20

**Authors:** Liubiana Arantes de Araújo

**Affiliations:** a Universidade Federal de Minas Gerais, Pós-graduação em Transtorno do Espectro do Autismo, Belo Horizonte, MG, Brazil; b Instituto de Neurodesenvolvimento BoraBrincar, Belo Horizonte, MG, Brazil

**Keywords:** Developmental disorder, Early signs, Cerebral palsy, Autism spectrum disorder

## Abstract

**Objective:**

To synthesize the most consistent warning signs (“red flags”) for NDDs: autism spectrum disorder, cerebral palsy, intellectual disability, language development disorder, coordination developmental disorder, attention deficit hyperactivity disorder, and global developmental delay.

**Data source:**

Review in PubMed/Medline, Cochrane Library, SciELO, CAPES, and BVS, 2003-2025. Methodological quality was assessed with NOS, CASP, and AMSTAR.

**Data summary:**

54 studies were included. The most consistent early signs were grouped by NPMD domains: Motor: delay to sit ≥ 9 m, absence of independent walking 18 m, absence of pincer grasp 10 m, and asymmetrical motor patterns 12 m; Language: absence of babbling at 9–12 m, lack of words until 15–18 m, and absence of two-word combination 24 m; Social: absence of social smile, poor eye contact, deficits in shared attention and communicative gestures; Cognitive and behavioral: regression of skills, repetitive behaviors, absence of symbolic play, and irritability or inattention; Others: atypical sensory responses, sleep disturbances, and feeding difficulties. Instruments such as M-CHAT-R/F, ASQ, Bayley, and HINE increased the accuracy of screening and reduced referral delays.

**Conclusion:**

Early recognition of warning signs for NPMD disorder associated with complementary examinations and formal assessment should be integrated into routine pediatric care.

## Introduction

Neurodevelopmental Disorders (NDDs) constitute a heterogeneous group of early-onset conditions characterized by deficits in the development of cognitive, motor, linguistic, socio-emotional, and adaptive functions, which interfere with individual functioning and social participation. These conditions result from alterations in the maturation process of the central nervous system and generally manifest in the first years of life, during critical periods of skill acquisition [1].

The diagnosis of NDDs is essentially clinical, carried out through direct observation of the child, interviews with parents or caregivers, and the application of standardized and validated instruments for screening and assessment. The internationally recognized diagnostic criteria are described in the International Classification of Diseases - 11^th^ ed. (ICD-11) [2], published by the World Health Organization (WHO), as well as in the Diagnostic and Statistical Manual of Mental Disorders - DSM-5-TR [3], which provide operational descriptions for each disorder, such as autism spectrum disorder (ASD), attention deficit/hyperactivity disorder (ADHD), communication and learning disorders, among others [2].

Advances in neuroscience have shown that early intervention in children with developmental delays is crucial for reducing long-term impairments. The infant brain exhibits high neuroplasticity and many windows of opportunity, critical periods in which specific neural circuits are more sensitive to environmental experiences and interventions, especially in the first years of life, meaning that neural connections can be strengthened or remodeled according to the received stimuli [4].

During early childhood, there are well-established windows for the development of language, vision, motor functions, and socio-emotional skills. Studies in neuroimaging and neurophysiology show that, during these periods, the brain undergoes intense formation of new connections, synaptic pruning, and myelination, processes that refine neural networks, making them more efficient [4]. When there is adequate stimulation, these circuits consolidate and define the learning of a particular skill; when there is a delay without intervention, damage can occur that is later difficult to reverse.

In this context, the childcare visit should be understood as a privileged space not only for monitoring physical growth, but also for the systematic assessment of neuropsychomotor development (NPMD). It is during these meetings that subtle signs should be detected, including delays in developmental milestones or even regressions - situations that require immediate attention from the professional who monitors the child.

It is important to emphasize that the identification of risk factors or early signs should lead to immediate intervention, even if a definitive diagnosis has not yet been made. Neuroscience shows that initiating interventions soon after exposure to a risk factor or after detecting any delay is to take advantage of periods of greater response due to neuroplasticity and windows of opportunity, and significantly increases the chances of functional gains [4].

In Brazil, Law N. 13,438/2017 establishes the mandatory formal assessment of child development in childcare visits within the Unified Health System (SUS). This must be done through the careful completion of the NPMD milestones section in the Child Health Booklet. In addition, the Brazilian Society of Pediatrics (SBP) recommends that all pediatricians apply the M-CHAT (Modified Checklist for Autism in Toddlers) at 18 and 24-month visits, favoring the early detection of warning signs for ASD [5,6].

In short, the responsibility for identifying risk factors, delays, or regressions lies with the person caring for the child. Given the importance of this topic, this study was designed to highlight the main warning signs for NDDs. It is the responsibility of pediatricians and other healthcare professionals to act vigilantly, initiating the intervention immediately, thus maximizing the possibility of transforming developmental trajectories.

## Methodology

A literature review was conducted aiming to identify warning signs for Neurodevelopmental Disorders (NDDs). Systematic and non-systematic reviews, quantitative, qualitative, and mixed-methods studies published in the BVS Portal (Virtual Health Library), SciELO (Scientific Electronic Library Online), Cochrane Library, CAPES Journals, and PubMed/Medline were included, in addition to secondary references extracted from the selected articles.

The formulation of the research question followed the PICO model (Patient, Intervention, Comparison, Outcome), structured as follows: Guiding question: "What are the warning signs for identifying Neurodevelopmental Disorders?"

The DeCS descriptors were applied to search strategies in the BVS and SciELO portals, while the MeSH descriptors were used to construct the strategy applied in PubMed/Medline according to the following combinations:

• "Neurodevelopmental Disorders" AND "Early Diagnosis" OR "Early Signs" OR "Red Flags"
• "Cerebral Palsy" AND "Early Signs" OR "Red Flags"
• "ADHD" AND "Early Signs" OR "Red Flags"
• "Developmental Coordination Disorder" AND "Early Signs" OR "Red Flags"
• "Global Developmental Delay" AND "Early Signs" OR "Red Flags"
• "Intellectual Disability" AND "Early Signs" OR "Red Flags"
• "Speech and Language Delay" AND "Early Signs" OR "Red Flags"
• "Autism Spectrum Disorder" AND "Early Signs" OR "Red Flags"

**Figure 1:**
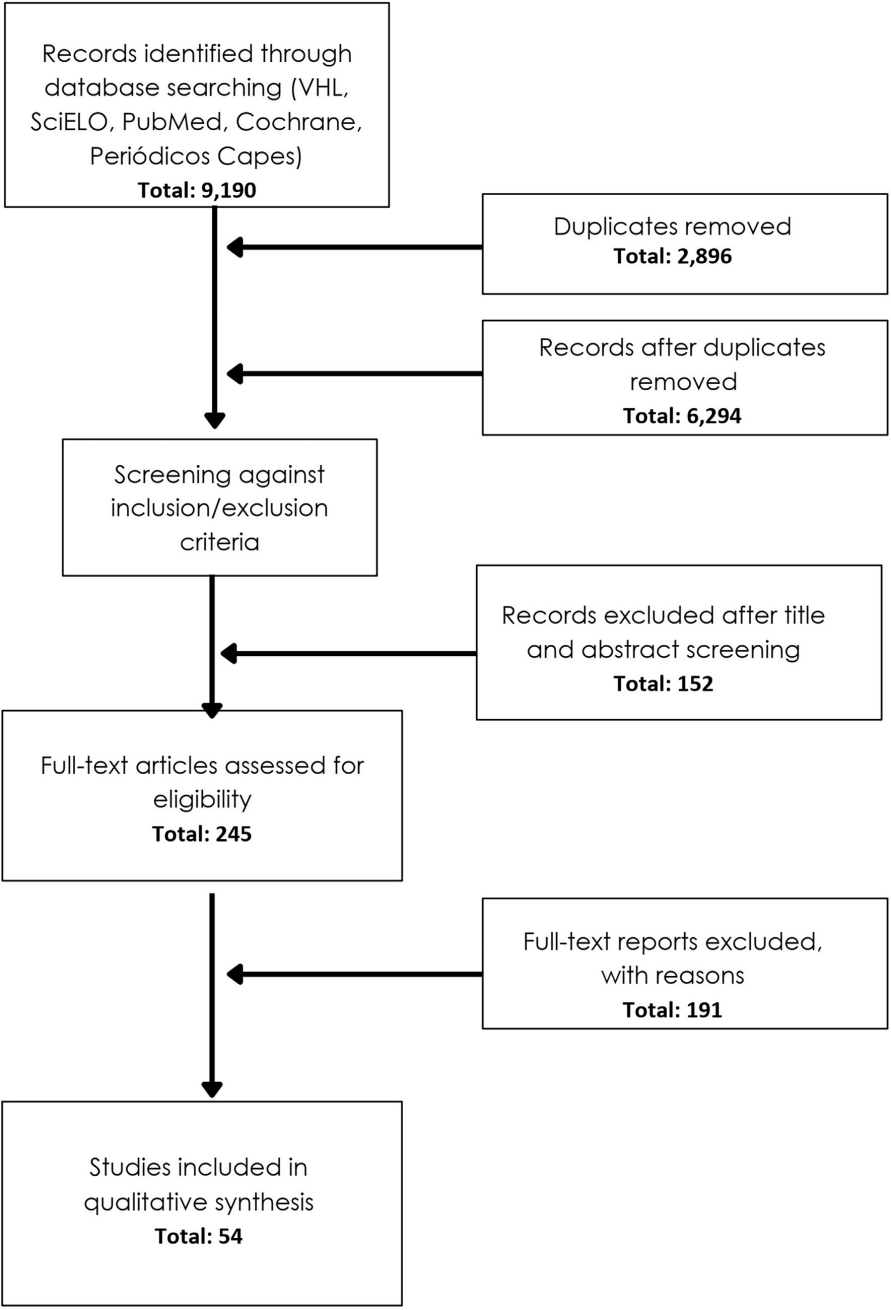
Flow chart.

The selected studies followed Flowchart 1 depicted in Figure 1.

The narrative synthesis was organized by clinical condition, aiming to facilitate the identification of the main early markers described in the literature for each NDD. Of the total, 20 studies were evaluated with validated methodological analysis instruments: 6 systematic reviews by AMSTAR 2, 9 observational studies by CASP, and 5 cohort or case-control studies by the Newcastle-Ottawa Scale (NOS).

## Results

Fifty-nine articles published in Portuguese, English, and Spanish, published between 1990-2025, involving humans and children up to 6 years of age, were included. Inclusion criteria were studies focusing on the description of warning signs for one of the studied NDDs, and exclusion criteria were case reports and case series.

The main focus of these studies was the evaluation of warning signs for GDD, CP, and ASD, in which the largest body of available scientific evidence is concentrated. In a complementary and less detailed manner, signs for attention deficit hyperactivity disorder (ADHD), intellectual disability (ID), developmental language disorder (DLD), and developmental coordination disorder (DCD) were described, recognizing their importance for clinical practice and pediatric surveillance (Table 1).

**Table 1:** Evaluation of the included studies according to AMSTAR 2, CASP and NOS.

**Reference**	**Type of study**	**Strong points**	**Limitations**	**Evaluation**
Law J et al., 2003[52]	Cochrane systematic review with pre-registered protocol.	Rigorous quality assessment (Cochrane Risk of Bias). Quantitative meta-analysis with heterogeneity analysis.	Probable restriction to articles in English. Does not detail search in grey literature.	HIGH AMSTAR
Nelson et al., 2006[13]	Systematic review	Structured search; clear outcomes	Did not assess bias in all cases; no meta-analysis; little grey literature.	MODERATE AMSTAR
Warren et al., 2016[18]	Systematic review (screening 1–4 years)	Protocol (PROSPERO CRD42014009809). Extensive search (MEDLINE, Embase, PsycINFO). Quality assessment. Critical discussion of the limitations.	Two heterogeneous studies were included. Language restriction (English/French). No detailed exclusion list. Publication bias not formally assessed.	HIGH-MODERATE AMSTAR
Peacock-Chambers et al., 2017[20]	Systematic review	Systematic approach and clear categorization of interventions. Recognition of methodological limitations and heterogeneity. Conclusions based on consistent primary care evidence.	No PROSPERO; Limited search (only PubMed and PsycINFO; no grey literature). Lack of confirmation of duplicate selection/extraction. Publication bias not formally assessed.	MODERATE-HIGH AMSTAR
Gomes et al., 2015[50]	Systematic review	Broad search without language restrictions. Quality assessment (CASP and AMSTAR). Critical discussion of methodological limitations. Relevant contextualization about Brazilian families.	No protocol registered. Selection/extraction not described in duplicate. Exclusion list incomplete. Publication bias not assessed.	MODERATE AMSTAR
Morgan et al., 2021[24]	Systematic review	Extensive search across 6 databases (CINAHL, Cochrane, Embase, MEDLINE, PsycINFO, Scopus). Quality assessment (AMSTAR/Cochrane RoB). Conditional recommendations based on the strength of evidence.	May not include grey literature. Not all exclusions listed. Publication bias not formally investigated.	HIGH-MODERATE AMSTAR
Sheldrick et al., 2011[10]		Use of quality criteria (QUADAS). Description of the characteristics of the studies. Critical discussion of limitations and heterogeneity.	Single database search (Medline). No pre-registration protocol. No explicit double selection. No publication bias analysis.	MODERATE AMSTAR
Araújo et al., 2021[9]	SYSTEMATIC REVIEW	Selection in duplicate. Good description of the included studies. Quality assessment (CASP and AMSTAR 2). Critical use of evidence and recognition of limitations.	Lack of publicly registered protocol. Potentially less comprehensive search. Non-transparent exclusion list. Unassessed publication bias.	MODERATE-HIGH AMSTAR
Maulik et al. 2011[47]	Meta-analysis	Extensive database (52 studies). Detailed description of samples and global variations. Use of random-effects model and subgroup analysis.	Absence of a registered protocol. Little detailed quality assessment of studies. Probable language/publication restriction. Publication bias not assessed.	MODERATE AMSTAR
Demirci & Kartal, 2016[11]	Cross-sectional (prevalence)	Adequate sample; valid instruments	Selection bias; poorly adjusted confounders	Moderate CASP
Kumar et al., 2024[19]	Cross-sectional (KAP parents)	Clear question; useful educational policies.	Selection bias; social desirability	Moderate/Low CASP
Barron-Garza et al., 2023[31]	Prospective cohort	Good follow-up; neuroimaging + clinical examination.	Partially adjusted confounders	Good CASP
Jain et al., 2024[32]	Diagnostic/prognostic cohort	Multicenter; clinical gold standard	Poorly described blindness	Good CASP
Romeo et al., 2013[28]	NICU Cohort	Standardized neurological examination	Referral bias; limited control	Moderate CASP
Gammer et al., 2015[36]	Prospective Cohort (ASD)	Standardized protocols; adequate follow-up.	Sample restricted to at-risk siblings	Good CASP
Ozonoff et al., 2011[39]	Multicenter cohort (ASD)	Robust sample; standardized protocols	Restricted generalization	High CASP
Elsabbagh et al., 2012[43]	Prospective cohort	Objective biomarker; diagnostic confirmation	Small sample size; limited spectrum	Good CASP
Aragão et al., 2017[29]	Cohort (Congenital Zika)	Well-defined cases; reliable neuroimaging.	No control group; poor adjustment for confounders.	Moderate NOS
Jones & Klin, 2013[42]	Prospective cohort (eye-tracking)	Robust cohort; paired controls; adequate follow-up	Limited adjustments of confounders	High NOS

## Global Developmental Disorder (GDD)

Global developmental disorder (GDD) is a heterogeneous condition characterized by significant delays in multiple domains – cognitive, linguistic, motor, and social. Children with GDD often show early signs that can be observed in pediatric consultations: absence of social smile by 3 months, inability to hold their head up by 4 months, inability to sit without support by 9 months, absence of babbling by 12 months, inability to walk independently by 18 months, or absence of meaningful words after 24 months. Population studies estimate a prevalence of 1-3% for global delays and up to 10-15% when considering delays in preschoolers [[7], [8], [9]]. Early detection is crucial, as interventions carried out in the first years take advantage of critical periods of brain plasticity. However, barriers to recognition persist, such as the low sensitivity of professionals who do not use validated instruments and the difficulty parents have in recognizing subtle delays [10]. It is worth highlighting that the COVID-19 pandemic increased exposure to risk factors, with a negative impact on children's development, and that maternal depression is an isolated risk factor for GDD [11].

A study conducted in primary care centers in Turkey demonstrated a prevalence of GDD of 6.4% among children aged 3 to 60 months, with the main signs being delayed crawling, walking difficulties, absence of simple phrases after 2 years, and poor social interaction. Significant associations were identified with advanced maternal age, low parental educational level, unfavorable socioeconomic conditions, and consanguineous marriages [11].

Complementarily, other authors have listed additional risk factors, such as poor maternal health during pregnancy, perinatal complications, infections, genetic predisposition, exposure to toxins, trauma, neglect, maltreatment, low socioeconomic status, family history of speech delay, male sex, and perinatal factors [12,13].

*Relevant warning signs for NDD include:*

• Motor and Language: delay in sitting, crawling, walking, or talking, compared to typical developmental milestones.
• Social Interaction: little eye contact, absence of social smile, difficulty sharing attention, limited imitation, low engagement.
• Repetitive Behaviors: intense and decontextualized repetitive hand or body movements, unusual use of objects, insistence on rigid routines.
• Play: poor functional, symbolic, and imaginative play; difficulty exploring toys in a varied way.
• Sensory, Attention, and Temperament: atypical responses to sensory stimuli (hypo/hypersensitivity), attention difficulties, and emotional regulation problems.
• Sleep and Feeding: persistent sleep problems or recurrent feeding difficulties.

The literature describes that the use of validated instruments, such as scales to assess different domains, increases early detection. The Ages and Stages Questionnaire (ASQ) identified children with motor delays and deficits in receptive and expressive language, and questionnaires applied to parents may have good acceptability (75–81% completeness) [14]. The Child Development Inventory (CDI) was more specific for the absence of phrases, difficulties in solving simple problems, and fine motor coordination deficits. The Parents’ Evaluation of Developmental Status (PEDS) highlighted recurring parental concerns, such as poor eye contact, attention difficulties, and repetitive behaviors [12].

The Bayley scale describes the following signs related to TND: absence of significant babbling at 12 months (expressive language); difficulties in verbal comprehension at 18 months (receptive language); not walking independently by 18 months (gross motor skills); persistent problems with pincer grasp or fine motor coordination after 12-15 months (fine motor skills); low engagement in simple cognitive problem-solving tasks after 18-24 months (cognitive) [15]. In Iran, the concurrent validity of the Bayley Screening Test version was evaluated in comparison to the full Bayley-III in 204 children aged 1 to 42 months. Strong correlations were found in all domains (r > 0.884), high specificity (87.8% to 100%), and higher sensitivity in receptive language (81.4%) and lower sensitivity in gross motor skills (58.1%), indicating that the screening version is useful but requires caution in detecting subtle motor delays [16]. In Suriname, a study analyzed the motor subtests of the Bayley-III in children aged 3 to 36 months, and the findings showed that such references did not adequately reflect local performance, with overestimation and underestimation of motor development. This result reinforces the need for cultural and regional validation [17].

In addition to technical limitations, caregivers' perception also interferes with early identification. Although almost 80% of parents expressed interest in screening, only 2.6% were aware of the availability of these services and signs such as lack of imitation, difficulties in pretend play, and atypical language use [18]. This means that warning signs such as persistent hypotonia, regression of acquired skills, absence of communicative gestures at 12 months, or unsteady gait may go unnoticed [18].

A study in Pakistan with 390 parents of children aged 0-5 years showed low knowledge about developmental milestones: 59% in gross motor skills, 54% in fine motor skills, 56% in the social domain, and 42% in language. Only 29% reported receiving adequate information from pediatricians, although 60% considered the delay a cause for concern. In case of suspicion, 55% would seek a general pediatrician, and a few specialists. Urban parents and parents from nuclear families demonstrated greater knowledge, reinforcing the need to expand parental education and the pediatricians’ engagement [19].

Hence, the discussion about structured programs increasing early referral rates (RR 1.95 in groups with office support and 1.71 in groups without support), in addition to reducing the time to intervention by up to 70% [20]. In the United States, a study demonstrated that early intervention led to significant gains in cognition, behavior, and language, including expanded vocabulary, better articulation, social advances, and a reduction in repetitive behaviors [20]. However, without validated instruments, the detection capacity of pediatricians is low, with sensitivity ranging from 0.14–0.54 [19].

In 2024, a team assessed whether there were changes in the average percentiles of child development over four decades, comparing data from 2,065 children between 0 and 36 months (1970s and 2018) and concluded stability of the milestones. However, the authors highlighted the importance of periodic reassessments of the scales [21].

## Cerebral palsy

Cerebral palsy is a group of permanent, but non-progressive, disorders of movement and posture development that cause limitations in motor activities, attributed to non-progressive changes in the developing brain, usually during the prenatal, perinatal period, or in the first years of postnatal life. It results from static brain injury that compromises structures related to motor control, such as the motor cortex, basal ganglia, cerebellum and their associated pathways. Clinical manifestations may include spasticity, dyskinesia (dystonia and choreoathetosis), ataxia, and are frequently accompanied by comorbidities such as intellectual disability, epilepsy, sensory disorders (visual and auditory), communication difficulties, gastrointestinal disorders and musculoskeletal alterations [22,23].

The diagnosis becomes more robust when combining a high-risk clinical history, structured neurological examination, assessment of spontaneous movements, and neuroimaging, rather than relying solely on motor delays observed late [22,24].

In the clinical examination, some neurological findings are particularly relevant, as described in Table 2.

**Table 2:** Main clinical signs suggestive of risk for cerebral palsy (0–12 months) [21,23,25].

Clinical finding	Description	Clinical implication
Poor cervical balance > 3–4 m; hyperextension/axial arching	Difficulty holding the head up, stiffness or arching of the torso.	Suggests a delay in postural control.
Closed fists > 3 m; postural asymmetry; hand preference < 12 m	Adducted thumb, clenched fists > 3 m, asymmetrical posture, hand preference < 12m	Early sign of hemiplegia
Persistent changes in muscle tone and primitive reflexes	Spastic hypertonia (scissoring pattern), hypotonia, persistent Moro reflex or ATNR > 5 m or absence of placing reflex.	Indicate impairment of the central motor pathways.
Feeding difficulties	Ineffective sucking, sucking-swallowing incoordination, drooling	Indicate bulbar involvement and risk of dysphagia.
Axial/proximal motor delay	No rolling, no forearm support, insufficient prone position, lack of upward vertical gaze.	Indicates deficit in axial strength and overall motor control, and cranial nerve involvement, related to dyskinesia.

Morgan et al. published a study used as a reference by the AAP in 2024; the data emphasize the importance of very early identification of CP, especially in high-risk newborns, such as extremely premature infants, those with hypoxic-ischemic encephalopathy, and those with perinatal strokes [24]. The authors emphasize that when using standardized, highly accurate tools such as the General Movements Assessment (GMA) and the Hammersmith Infant Neurological Examination (HINE), it is already possible to reach a diagnosis or at least classify the child as "high risk for CP" at around 3 months of corrected age, achieving sensitivity and specificity greater than 90% [22].

The GMA observes the quality of the baby's spontaneous movements through short videos (3-5 minutes) in calm wakefulness. It has high predictive value by identifying cramped-synchronized patterns in the first weeks or the absence of fidgety movements between 9 and 20 weeks post-term, considered one of the most sensitive and specific predictors for CP. It is a non-invasive, low-cost examination applicable in different contexts, although it depends on training and standardization of the recording.

The HINE, in turn, is a structured clinical examination applied between 2 and 24 months of age, which assesses cranial nerves, posture, tone, voluntary movements, postural reactions, primitive reflexes, and behavior. The score ranges from 0 to 78, with values below 57 between 3 and 6 months being strongly associated with CP and also allowing the characterization of the motor subtype. Its application is quick (10–15 minutes), low-cost, and reproducible, but requires training to ensure standardization [24,25] (Table 3).

**Table 3:** Comparison between GMA and HINE.

**Characteristic**	**General Movements Assessment (GMA)**	**Hammersmith Infant Neurological Examination (HINE)**
Age range	0 to 20 weeks post-term (especially 9–20 weeks for “fidgety movements”)	2 to 24 months
Main objective	Assess the quality of the baby's spontaneous movements.	To evaluate the infant’s neurological examination in a structured way.
Method	Video observation (3–5 min), with the baby awake and calm, supine position.	Standardized clinical examination (10–15 min) with a score (0–78 points)
Main parameters evaluated	Variability, fluidity, and complexity of movements; presence or absence of "fidgety movements"	Cranial nerves, posture, tone, voluntary movements, postural reactions, primitive reflexes, behavior
Risk signs for CP	Absence of fidgety movements between 9–20 weeks has high predictive value for CP.	A score < 57 at 3–6 months is strongly associated with cerebral palsy; asymmetries and persistent reflexes reinforce suspicion.
Advantages	Non-invasive, low cost, high sensitivity and specificity, applicable early.	Fast, structured, quantifiable, allows monitoring of progress, good inter-observer reliability.
Limitations	Requires specialized training for video analysis; depends on ideal recording conditions.	Requires training for scoring; depends on the baby's cooperation during the examination.
Predictive value	One of the most sensitive methods for CP in infants under 5 months.	Strong predictor of CP and useful for functional stratification; combined with GMA it increases diagnostic accuracy.

This proposal breaks with the traditional practice of waiting for evident motor delays only in the second year of life, advocating the adoption of screening protocols with a positive impact on the child's motor and cognitive prognosis and on the well-being of families [22].

Observational studies reinforce the value of early recognition of clinical signs in the detection of CP, and the absence of the Moro reflex and plantar grasp in at-risk infants can predict adverse outcomes, including CP [25]. Retention of primitive reflexes may be associated with motor delay in children with CP or correlate with motor and postural difficulties [26,27]. Romeo et al. confirmed that HINE subscores robustly stratify risk [28]. In cases such as congenital Zika syndrome, the persistence of multiple primitive reflexes and the absence of the parachute reaction were associated with worse motor outcomes [29].

Cerebral magnetic resonance imaging and transfontanellar ultrasound increase diagnostic accuracy when they reveal typical lesions, such as periventricular leukomalacia (PVL) and cortical infarcts [22].

Prospective studies confirm the clinical applicability of standardized instruments for early detection of CP. In the United Kingdom, Marcroft et al. followed 95 extremely premature infants until 2 years of corrected age; 13 (13.7%) were diagnosed with CP. GMA performed between 11 and 18 corrected weeks showed the highest accuracy (sensitivity of 92.3% and specificity of 98.9%), surpassing LAPI and cranial ultrasound (cUS), which did not add predictive value to GMA [30].

A study conducted in Mexico with children up to 18 months old found an incidence of 4.4/1000 live births - higher than the average of 2.0-2.5/1000 in developed countries. The most common early clinical signs included poor head control after the fourth month, persistent clenched hands, postural asymmetry, and early hand preference. Feeding difficulties, such as ineffective sucking and choking, were also observed. Neuroimaging showed PVL as the most frequent lesion, followed by intraventricular/subependymal hemorrhage and cortical atrophy associated with ventricular dilation [31].

The CINEPS multicenter study evaluated 395 preterm infants with ≤ 32 weeks of gestation using sMRI between 39-44 weeks, GMA, and HINE between 12-18 weeks. At 2 years, 39 children (11.5%) were diagnosed with CP, mostly Gross Motor Function Classification System (GMFCS) level I (28 cases). The combination of sMRI with GMA had a specificity of 100% and a sensitivity of 22%; sMRI with HINE had a sensitivity of 32% and a specificity of 98%. For moderate to severe cases (levels II–V), sensitivity ranged from 78% to 100%, but remained low for mild cases, highlighting the limitations of these tools in the early detection of more subtle cases [32].

Interpreting PVL as an etiological factor requires caution since normal variants and genetic diseases can mimic its characteristics in neuroimaging [33].

Recent studies with artificial intelligence (AI) and machine learning (ML) have demonstrated the potential to detect subtle motor changes that are not yet visible in clinical examination, such as deviations in amplitude and motor coordination. These algorithms can complement methods such as GMA, increasing the screening sensitivity and allowing timely interventions during the critical window of neuroplasticity [22,33].

In summary, the association between early clinical signs, standardized tools, and neuroimaging allows a faster diagnosis of cerebral palsy, favors early interventions, and improves functional outcomes.

## Developmental Disorder

Developmental disorder is an umbrella term that encompasses any condition in which a child development does not follow the expected course, whether in language, cognition, socialization, behavior, or motor skills [2,3].

*Examples (according to DSM-5-TR and ICD-11):*

• ASD (Autism Spectrum Disorder)
• ADHD (Attention Deficit Hyperactivity Disorder)
• Communication Disorders (speech, language, pragmatics)
• Motor Coordination Disorders
• Intellectual Disability
• Specific Learning Disorders

## Autism Spectrum Disorder (ASD)

Autism Spectrum Disorder (ASD) is a neurodevelopmental disorder characterized by a set of persistent deficits in communication and social interaction, associated with restricted and repetitive patterns of behavior, interests, or activities, manifesting from the early developmental period [3].

The diagnosis is based on persistent deficits in communication and social interaction across a range of contexts, including difficulties in socio-emotional reciprocity, nonverbal communicative behaviors, and the development or maintenance of relationships; and restricted and repetitive patterns of behavior, interests, or activities, manifested by stereotyped movements, insistence on routines, fixed interests, and/or alterations in sensory reactivity. These symptoms must be present from early development and cause significant impairment in overall functioning [2,3].

Symptom onset typically occurs in early childhood, although full clinical manifestation may only become evident when social demands exceed individual capabilities. Severity is determined by the intensity of deficits and functional impact, being heterogeneous - ranging from level 1, compatible with relative independence, to level 3, requiring substantial support in multiple contexts [2,3].

Thus, ASD is not defined by a single marker, but by a dimensional and continuous pattern, reflecting the interaction between genetic predisposition, neurobiological and environmental factors, and characterized by a wide variability of clinical, cognitive, and adaptive profiles.

In the last two decades, the prevalence of ASD has shown marked growth, due to greater awareness, advances in screening methods, changes in diagnostic criteria, and an increase in the number of cases. Global prevalence studies estimate the prevalence to be 1/100 [34]. In 2000, surveys by the Autism and Developmental Disabilities Monitoring (ADDM) indicated a prevalence of 1/150; 1/88 in 2008, 1/68 in 2014, 1/54 in 2020, and CDC data from 2025 show that 1/31 children have ASD[35] ASD is no longer considered rare and has become an important public health challenge, requiring an organized and integrated response in the clinical, educational, and social fields.

Early recognition of warning signs is crucial to expanding opportunities for intervention in time before windows of opportunity close. The scientific literature points out that the most consistent signs include shared attention deficit, considered the most robust marker, lack of response to name, absence of communicative gestures - especially declarative ones -, delay in expressive language, impoverished symbolic play, reduced sustained gaze, in addition to the presence of repetitive and restrictive behaviors (RRBs) of atypical quality and frequency and preference for objects over human faces[36] (Table 4).

**Table 4:** Risk signs for ASD.

**Early warning sign (red flag)**	**Expected age in typical development**	**Change observed in ASD**
Shared attention deficit	9–12 months: follows gaze/gesture; 12–18 months: alternates gaze.	Absence of eye contact, does not point to share, does not alternate gaze between object/caregiver.
No response to name	9–12 months: responds consistently	Repeated failure to respond, despite preserved hearing.
Absence of communicative gestures	< 12m: “goodbye”, show; 12–15m: point; 16m: ≥ 16 gestures	Absence of "goodbye" at 12 months, no declarative pointing (12–18 months), poor gestural repertoire at 16 months.
Expressive language delay	16m: isolated words; 24m: 2-word phrases	No words by 16 months or no 2-word phrases by 24 months; delay associated with social deficits.
Reduced sustained gaze	From 2–3 months: responsive eye contact	Progressive decrease in attention during eye contact between 2–6 m; inconsistent eye contact.
Repetitive and restrictive behaviors	6–24m: transient repetitive movements	Persistence, high intensity, inflexibility, functional impact; replaces symbolic play.
Preference for objects	6–12 months: natural preference for human faces	Increased fixation on geometric objects/patterns rather than faces.
Biomarkers (Early Point, Eye Tracking)	12–18m: declarative pointing; 2–6m: eye fixation	Absence of declarative pointing; early decline in eye attention; increased focus on objects.
Loss of acquired milestones	Continuous skill progression is expected.	Regression: loss of previously acquired words, gestures, or eye contact.
Standardized screening (M-CHAT-R/F, CSBS-DP)	18 and 24 months (AAP recommends universal application)	Identifies subtle signs and increases the chance of early detection.

In recent years, advances such as Early Point and eye tracking have stood out as potential objective biomarkers, capable of anticipating identification even before the full emergence of clinical manifestations [37,38].

Among all these signs, shared attention deficit occupies a prominent place. In typical development, it is expected that between 9-12 months, babies will follow the gaze or gesture of an adult, that at 12 months they will begin to point both to ask (proto-imperative) and to share interest (proto-declarative), and that between 12-18 months they will alternate their gaze between object and caregiver, consolidating social reciprocity. Gestures precede verbal language and function as scaffolding for the acquisition of speech. In typical babies, conventional gestures such as "bye-bye" and the act of showing objects already appear before 12 months, and at 16 months, a minimum diversity of 16 gestures is expected. In children with ASD, these behaviors tend to be absent, delayed, or qualitatively impoverished. Bryson et al. observed deficits as early as 12 months in at-risk siblings, while Gammer et al., applying the Autism Observational Scale for Infants (AOSI), identified early deficits in babies who later met the criteria for ASD [34,36]. The absence of shared attention before 18 months has high predictive value for diagnosis, and this domain is one of the best predictors of language development and social competence [37]. In line with this, the AAP and SBP recommend that its absence be considered an essential red flag in the 18- and 24-month consultations [38].

In autism, the absence of the "bye-bye" gesture is frequently observed around 12 months, as well as declarative pointing between 12 and 18 months, considered one of the main red flags and the basis of the Early Point concept [37]. Prospective studies with at-risk siblings reinforce that the lack or delay in gestures differentiates early on those who evolve into ASD [34,37].

Another widely studied marker is the absence of response to name between 9 and 12 months [37,39]. Non-response to name may correlate with worse adaptive outcomes at 3 years. Thus, this sign has become a priority in screening protocols [40].

Expressive language delay is also a critical marker, being one of the most consistent and reported red flag signs for ASD. Two milestones stand out: absence of single words by 16 months and absence of spontaneous two-word phrases by 24 months, and when associated with social deficits, it increases the accuracy for ASD[13] Lord et al. reinforced that this absence, especially in conjunction with the lack of declarative gestures, is highly predictive [41].

Responsive and sustained eye contact is one of the first indicators of social development in babies, appearing in the first months of life, and in children with ASD, this marker is diminished or inconsistent from the first year, hindering the construction of shared attention and communicative engagement [42].

Eye-tracking studies show that babies who were later diagnosed with ASD have a progressive decline in eye fixation between 2 and 6 months. Jones and Klin identified that this early decrease in attention to human gaze was one of the most consistent precursors of ASD [42], while Elsabbagh et al. confirmed that at-risk siblings have face orientation deficits before the full manifestation of symptoms [43].

Repetitive and restrictive behaviors, although also present in typical children, take on distinct characteristics in ASD. In these children, they emerge at the end of the first year and become more intense in the second, becoming persistent, inflexible, and with functional impact, replacing symbolic play and hindering social interaction [[44], [45], [46]].

Finally, visual interest in objects to the detriment of human faces constitutes another early marker. Children between 6 and 12 months old who developed ASD exhibited less fixation on human faces and greater focus on non-social elements, and those who prefer geometric patterns are more likely to have ASD, in addition to being associated with greater clinical severity [6,7,47].

Extreme food selectivity and sensory disturbances are observed from the first year of life and can be considered signs of ASD [48].

A possible biological marker associated with the risk of ASD is the accelerated growth of head circumference after 3-4 months of age. Longitudinal studies have shown that some individuals who are later diagnosed with ASD exhibit a disproportionate acceleration of head circumference during the first year, especially between 4-12 months. This rapid increase may reflect alterations in neuronal proliferation, synaptogenesis, and synaptic pruning processes, resulting in atypical patterns of brain connectivity. Although the finding is not specific and should not be used alone as a diagnostic criterion, its presence, when associated with early clinical signs such as shared attention deficits and absence of communicative gestures, reinforces the need for surveillance and in-depth investigation [49].

Families have a low rate of detection of signs of autism and face many challenges in Brazil for this identification, diagnosis, and timely intervention [50]. In this context, it is crucial to highlight the role of standardized screening tests, such as the Modified Checklist for Autism in Toddlers, Revised with Follow-Up (M-CHAT-R/F), recommended by the AAP for universal application at 18 and 24 months [38]. The M-CHAT-R/F is especially useful for identifying subtle signs in primary health care settings, allowing at-risk children to be referred early for specialized evaluation. In addition to the M-CHAT-R/F, other instruments such as the CSBS-DP and observational scales complement screening in at-risk populations [38,51].

Another essential point is that, at any age, the loss of previously acquired developmental milestones should be considered an immediate red flag, requiring detailed investigation. Regression of skills, such as loss of words, gestures, or eye contact, substantially increases the clinical suspicion of ASD and other neurodevelopmental conditions, and cannot be attributed to benign individual variations [51].

In summary, early warning signs of ASD encompass deficits in multiple domains – social, communicative, linguistic, and behavioral – which, when assessed in an integrated manner, allow for greater sensitivity in clinical screening. The combination of classic behavioral markers (shared attention, gestures, name response, language, and gaze), emerging biomarkers (Early Point and eye tracking), and standardized screening protocols (M-CHAT-R/F) represents the most promising strategy for early identification and timely referral for intervention, in line with Neuroscience recommendations.

## Intellectual Disability

Intellectual disability is defined by cognitive and adaptive deficits with onset during the developmental period [2,3]. It involves impairments in reasoning, problem-solving, academic learning, and social skills, which compromise autonomy and can be measured through Intelligence Quotient tests, such as the SON-R and the WISC. Early signs indicative of ID are:[2,3]

• 0–6 months
  1. Delayed social smile.
  2. Little interest in faces or environmental stimuli.
  3. Persistent hypotonia.
• 6–12 months
  1. Delay in maintaining a sitting position.
  2. Poor imitation of sounds or expressions.
  3. Difficulty maintaining eye contact.
  4. Absence of babbling until 9 months.
  5. Not responding to name after 9 months.
• 12–18 months
  1. Absence of independent walking until 18 months.
  2. Absence of meaningful words until 15–18 months.
  3. Difficulty exploring objects functionally.
  4. Restricted vocabulary.
  5. Difficulty understanding simple commands.
• 18–24 months
  1. Excessive dependence on simple activities (feeding, dressing, manipulating toys).
  2. Difficulty with pincer grasp or manipulating small objects.
  3. Delay in autonomy (feeding, dressing, toilet training).
• Preschool
  1. Delay or absence of symbolic play.
  2. Difficulty with simple rules of social games.
  3. Slurred speech, lexical restrictions, grammatical limitations.
  4. Increased dependence on personal care.
• Domains of global developmental delay as risk markers for ID
  1. Language/Communication: absence of babbling until 9 months; not responding to name after 9 months; absence of words until 15-18 months.
  2. Cognition: difficulty imitating gestures, solving simple problems, or little interest in exploring the environment.
  3. Fine Motor Skills: difficulty manipulating small objects (e.g., pincer grasp).
  4. Gross Motor Skills: delay in rolling over (>6 months), sitting (>9 months), crawling (>12 months), walking (>18 months).
  5. Socio-adaptive: reduced eye contact, delay in autonomy (feeding, dressing).

The more severe the ID, the sooner global delays become evident, often as early as the first year of life. Thus, Global Development Delay (GDD) should be interpreted as a major red flag, requiring detailed neurological evaluation [47].

At any age, the loss of previously acquired milestones should be considered a warning sign for ID, ASD, or other neurological conditions. The application of screening instruments such as ASQ, PEDS, Bayley, and, for autism, the M-CHAT-R/F, substantially increases the chance of early detection [12,13]. Early intervention has a positive impact on cognition, language, and behavior, reinforcing the importance of early suspicion and referral [8,20].

## Language Development Disorder (LDD)

Language Development Disorder (LDD) is characterized by persistent difficulties in the acquisition and use of language, not explained by intellectual disability, ASD, hearing loss, or environmental deprivation. They can affect phonology, vocabulary, grammar, or pragmatics, interfering with functional communication [52].

The signs can be observed from the first months of life: between 6-10 months, the absence of canonical babbling, little varied vocalizations, and low responsiveness to the environment stand out; from 12-18 months, the absence of meaningful words and the scarce use of communicative gestures, such as pointing or waving goodbye; around 18 months, a restricted vocabulary and difficulty in understanding simple commands; at 24 months, the absence of spontaneous combination of two words; and after 3 years, poorly intelligible speech, lexical restrictions, grammatical and syntactic limitations remain [52].

These signs are also systematized in validated scales. The Early Language Milestone Scale (ELM), applied from 0 to 36 months, screens for delays in expressive, receptive, and auditory-visual language, highlighting as risk markers the absence of babbling up to 12 months, restricted vocabulary at 18 months, lack of word combination up to 24 months, and failures to understand simple commands. The ABFW - Test of Child Language, aimed at children aged 3 to 12 years, details vocabulary, phonology, fluency, and pragmatics, revealing in children with LDD a reduced vocabulary, the presence of atypical phonological processes beyond their age, fragile discursive coherence, and subtle pragmatic difficulties [52].

Thus, both the early signs observed clinically and standardized instruments such as ELM and ABFW reinforce the importance of screening and early referral for medical, auditory, and speech-language pathology intervention.

## Developmental Coordination Disorder (DCD)

DCD involves persistent motor difficulties that interfere with daily living and academic activities, not justified by cerebral palsy, neuromuscular diseases, or intellectual disability [53]. The literature describes the following early signs:

• 6–9 months: delay in sitting without support, frequent falls in sitting position, poorly coordinated movements.
• 12 months: difficulty crawling or supporting oneself to stand; unsteady gait upon onset.
• 18 months: delay or immaturity in walking; difficulty manipulating simple objects, such as blocks or spoons.
• 2–3 years: frequent stumbling, difficulty climbing stairs, playing with toys, kicking or throwing a ball, riding a tricycle.
• Preschool: difficulty holding a pencil, drawing simple shapes, cutting paper, or dressing independently.

## Attention Deficit Hyperactivity Disorder (ADHD)

ADHD is defined by a persistent pattern of inattention, hyperactivity, and impulsivity that causes impairment in two or more settings, with onset before age 12. Although the diagnosis can only be established after age 4, early signs of risk can be detected in the NPMD [2,3,54].

*Early signs in the NPMD*:[54,55]

• 0–6 months: marked irritability, sleep difficulties, high reactivity to environmental stimuli.
• 6–12 months: difficulty maintaining sustained visual attention on toys or social interactions.
• 12–24 months: excessive motor activity, difficulty remaining seated on laps, rapid and disorganized toy changes, frequent falls.
• Preschool (3–5 years): marked impulsivity (not waiting for turns, interrupting playtime), low frustration tolerance, difficulty maintaining focus on directed activities, frequent accidents due to impulsive behavior.

It is essential to differentiate between inadequate routines, such as lack of stimulation, toxic stress, or excessive screen time, which can cause NPMD delays, and cases of neurodevelopmental disorders, such as ADHD or ASD.

It is emphasized that in the presence of risk factors for any developmental disorder, early intervention is recommended; that is, even before showing any signs or delays, the child will benefit from stimulation to reach milestones. In the case of detecting warning signs of autism or ADHD, for example, intervention may no longer be considered early because a delay is already present, but it is still valid to intervene in time before the windows of opportunity close, taking advantage of the period of maximum neuroplasticity.

## Study limitations

This review has some limitations that should be considered. There is a risk of **publication bias**, since the search only included consolidated databases, and there may have been a loss of unpublished or difficult-to-access studies, which could overestimate the available evidence. In addition, there is **selection bias** because, despite the use of structured descriptors (DeCS/MeSH), the choice of studies may have included heterogeneous and non-comparable evidence. The **heterogeneity of the studies** also constitutes an important limitation, since they varied in methodological design, sample size, age of children, and diagnostic criteria, making quantitative synthesis and direct comparability difficult. Another aspect is the risk of **generalization**, since the studies that were analyzed came from distinct cultural and socioeconomic contexts without adequate stratification. Finally, there was a **lack of data for subgroup analysis**, as differences in early signs according to sex, prematurity, perinatal risk factors, family history, and early and inappropriate exposure to screens and toxic stress were not explored, which could refine the clinical applicability of the findings. These data suggest critical interpretation, but the methodological care in the search and application of **quality assessment** using validated instruments such as the Newcastle-Ottawa Scale, CASP, and AMSTAR is noteworthy. This information does not invalidate the robustness of the warning signs highlighted in this review and their applicability by physicians who monitor children so that they can reach their maximum developmental potential.

## Conclusion

Early recognition of warning signs for neurodevelopmental disorders is crucial for modifying functional trajectories and reducing long-term impairments. The literature demonstrates that early clinical markers, when associated with standardized screening instruments, substantially increase diagnostic accuracy and allow timely interventions during critical periods of brain plasticity. For pediatric practice, this implies systematic monitoring of developmental milestones, screening, and the routine use of validated scales, as well as immediate or timely intervention in the face of any risk factor, delay, or regression. However, significant gaps remain, such as the low sensitivity of isolated clinical screening and the need for greater cultural validation of available tools. In the coming years, advances in objective biomarkers and artificial intelligence should expand the capacity for early screening and diagnosis, consolidating early detection as a central axis to help at-risk children reach their best developmental potential.

## Financial support

None declared.

## Data availability statement

The author declares that the data are available upon request.

## Conflicts of interest

The author declares no conflicts of interest.
